# Population dynamics of epidemic and endemic states of drug-resistance emergence in infectious diseases

**DOI:** 10.7717/peerj.2817

**Published:** 2017-01-10

**Authors:** Diána Knipl, Gergely Röst, Seyed M. Moghadas

**Affiliations:** 1 Department of Mathematics, University College London, London, United Kingdom; 2 Bolyai Institute, University of Szeged, Szeged, Hungary; 3 Agent-Based Modelling Laboratory, York University, Toronto, Canada; 4 MTA-SZTE Analysis and Stochastic Research Group, University of Szeged, Szeged, Hungary

**Keywords:** Drug-resistance, Delay treatment, Epidemic and endemic states, Reproduction numbers

## Abstract

The emergence and spread of drug-resistance during treatment of many infectious diseases continue to degrade our ability to control and mitigate infection outcomes using therapeutic measures. While the coverage and efficacy of treatment remain key factors in the population dynamics of resistance, the timing for the start of the treatment in infectious individuals can significantly influence such dynamics. We developed a between-host disease transmission model to investigate the short-term (epidemic) and long-term (endemic) states of infections caused by two competing pathogen subtypes, namely the wild-type and resistant-type, when the probability of developing resistance is a function of delay in start of the treatment. We characterize the behaviour of disease equilibria and obtain a condition to minimize the fraction of population infectious at the endemic state in terms of probability of developing resistance and its transmission fitness. For the short-term epidemic dynamics, we illustrate that depending on the likelihood of resistance development at the time of treatment initiation, the same epidemic size may be achieved with different delays in start of the treatment, which may correspond to significantly different treatment coverages. Our results demonstrate that early initiation of treatment may not necessarily be the optimal strategy for curtailing the incidence of resistance or the overall disease burden. The risk of developing drug-resistance in-host remains an important factor in the management of resistance in the population.

## Introduction

The evolution of drug-resistance in many infectious diseases has proven to be one of the most challenging problems of human health in modern medicine. While a number of evolutionary mechanisms are generic for the rise of resistance, there are several processes which are specific to the drugs and treatment regimens (Zur Wiesch et al., 2011). These processes are often characterized by the competition between the resistant and wild pathogen subtypes. If no resistant-type exists before the start of treatment, the likelihood of an emergent resistant mutant out-competing the wild-type and dominating the pathogen population during treatment depends greatly on its fitness advantage over the wild-type (Lipsitch, 2001; Zur Wiesch et al., 2011). For a sufficiently low replicative fitness, the resistant-type may still be out-competed by the wild-type, even under a strong selection pressure of drugs (Ferguson et al., 2003; Moghadas, 2008). However, if the difference between the intrinsic fitness of the two pathogen subtypes is sufficiently small, then the selection pressure of drugs can overturn the competitive dynamics in favour of the resistant-type (Lipsitch, 2001; Ferguson et al., 2003; Moghadas, 2008; Zur Wiesch et al., 2011).

Within the fitness landscape of the pathogen subtypes, a key factor determining the competitive dynamics between the resistant-type and wild-type is the probability of developing resistance, which may be affected by the time for start of the treatment. For example, in influenza infection and in the absence of resistant mutants, early treatment inhibits viral replication, which will therefore minimize the likelihood of evolving resistant mutants. However, when resistant mutants are present, early treatment can provide an opportunity for the outgrowth of such mutants under the pressure of drugs (Alexander et al., 2007; Handel, Longini Jr & Antia, 2007). In this context, a major barrier to this growth is the generation of within-host adaptive immune responses. It has been shown that drug resistance is less likely to develop if the immune responses are maintained above a certain threshold during treatment (Lloyd & Wodarz, 2006; Wodarz, 2001; Wodarz & Lloyd, 2004), suggesting that a delay in start of the treatment may be beneficial in preventing resistance emergence given the timelines for developing immune responses (Wodarz, 2001). Acquisition of resistance during treatment is thus affected not just by the fitness of resistant-type, but also by the probability of resistant mutants occurring, which may be viewed as a function of delay in the initiation of treatment.

Previous studies illustrate how treatment coverage and efficacy influence the population dynamics of drug-resistance spread, while considering the fitness of resistant-type but leaving out the possibility of time-dependent probability of resistance development (Alexander et al., 2007; Huijben et al., 2013; Lipsitch et al., 2007; Legros & Bonhoeffer, 2016; Moghadas, 2008; Moghadas et al., 2008; Xiao, Brauer & Moghadas, 2016). Here we consider this probability as a function of delay in start of the treatment and discuss both short-term (epidemic) and long-term (endemic) states of the resistant-type and with-type infections. For the endemic state, we characterize the behaviour of disease equilibria and obtain a condition to minimize the fraction of population infectious at equilibrium with a delay in start of the treatment within the infectious period. For the epidemic state, we show that in contrast with previous work on the short-term disease dynamics (Lipsitch et al., 2007; Moghadas, 2008; Moghadas et al., 2008; Xiao, Brauer & Moghadas, 2016), the minimum epidemic size may be achieved with different treatment coverages (i.e., the fraction of infected population treated) depending on the functional form of the probability of resistance development.

## Methods

### The model

We consider a population that is fully susceptible to the invading pathogen. The model includes two pathogen subtypes, namely, the wild-type and resistant-type. We assume that the resistant-type may prevail during the treatment of individuals infectious with the wild-type. The probability of developing resistance is assumed to be a function of delay in start of the treatment, and we assume that this delay is the same for everyone in the population. To describe the transmission dynamics between susceptible and infectious individuals, we use a mass-action incidence in a homogeneously mixing population, and assume that the resistant-type emerges with a relative transmission fitness (*δ*) compared to that of the wild-type, where *δ* = 1 describes the absence of fitness advantage or disadvantage for direct transmission of resistance. For many diseases (e.g., influenza), resistance generally emerges with a negative fitness advantage (*δ* < 1); however, fitness costs of resistance can be ameliorated with compensatory mutations (Handel, Regoes & Antia, 2006; Rimmelzwaan et al., 2005). There are also diseases (e.g., tuberculosis) for which resistance may initially emerge with a positive fitness advantage (*δ* > 1) (Borrell & Gagneux, 2009; Casali et al., 2014). In the model presented here, we consider a delay *τ* in the treatment initiation during the infectious period, and denote the delay-dependent probability of developing resistance by *q*(*τ*). We also assume that the treatment is ineffective against the resistant-type infection.

Denoting the class of susceptible individuals by *S*, and the classes of individuals infectious with the wild-type and resistant-type by *I*_*w*_ and *I*_*r*_, respectively, the dynamics of disease propagation is governed by the time-dependent differential equations system:

(1) \begin{eqnarray*}\begin{array}{@{}l@{}} \displaystyle {S}^{{^{\prime}}}(t)=\mu -\beta S(t)[{I}_{w}(t)+\delta {I}_{r}(t)]-\mu S(t),\\ \displaystyle {I}_{w}^{{^{\prime}}}(t)=\beta S(t){I}_{w}(t)-\beta S(t-\tau ){I}_{w}(t-\tau ){e}^{-(\mu +\gamma )\tau }-\gamma {I}_{w}(t)-\mu {I}_{w}(t),\\ \displaystyle {I}_{r}^{{^{\prime}}}(t)=\delta \beta S(t){I}_{r}(t)+q(\tau )\beta S(t-\tau ){I}_{w}(t-\tau ){e}^{-(\mu +\gamma )\tau }-\gamma {I}_{r}(t)-\mu {I}_{r}(t),\\ \displaystyle {T}^{{^{\prime}}}(t)=(1-q(\tau ))\beta S(t-\tau ){I}_{w}(t-\tau ){e}^{-(\mu +\gamma )\tau }+\gamma [{I}_{w}(t)+{I}_{r}(t)]-\mu T(t). \end{array}\end{eqnarray*}

where 1∕*μ* is the average lifetime of the host population, and 1∕*γ* is the average infectious period (assumed to be the same for both wild-type and resistant-type infections). Here, we omit the disease-induced death rate and assume that the disease has no effect on the average lifetime of the infectious individuals. A more general case where the average lifetime of the infectious individuals differs from that of the healthy individuals is considered in the Supplemental Information.

**Figure 1:**
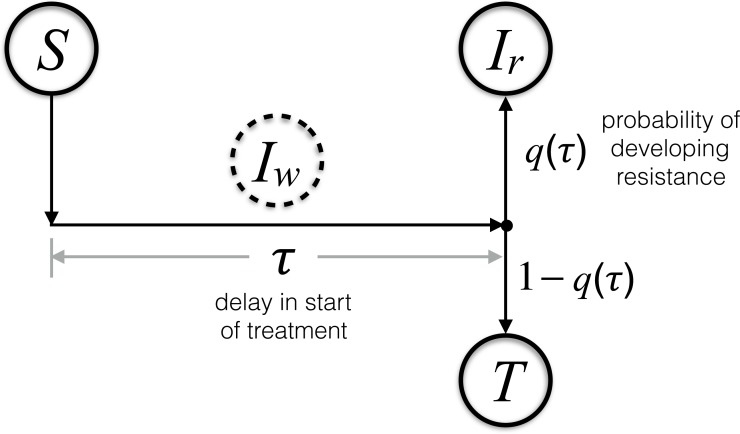
Schematic diagram of the transitions between model compartments.

In this model, we assume that each infectious individual will receive treatment precisely *τ* units of time after becoming infected. The term *βS*(*t*)*I*_*w*_(*t*) gives the cohort of individuals who enter the class *I*_*w*_ at any time *t*. Since the times until recovery and death are exponentially distributed with means 1∕*γ* and 1∕*μ*, respectively, the probability for an individual to remain in the *I*_*w*_ class *τ* units of time after entering the class is *e*^−(*μ*+*γ*)*τ*^. Thus, the transition out of the *I*_*w*_ class by means of treatment at any time *t* can be described by the term *βS*(*t* − *τ*)*I*_*w*_(*t* − *τ*)*e*^−(*μ*+*γ*)*τ*^, accounting for the cohort of individuals who were infected by the wild-type pathogen at time *t* − *τ* and therefore receive treatment at time *t* (i.e., *τ* units of time after becoming infected). Treated individuals will either develop resistance with the probability *q*(*τ*) (and move to the *I*_*r*_ class), or become effectively treated (and move to the *T* class). Since the treatment is ineffective against resistance, for simplicity we included treated and untreated individuals infectious with the resistant-type in the same class *I*_*r*_ (Fig. 1). Lastly, the class *T* represents all individuals who are recovered from infection or effectively treated.

A careful bookkeeping of transitions between the compartments allows us to describe the dynamics by a system of differential equations, presented in (1). As described above, the system dynamics at any time *t* is not only determined by the present number of individuals in each compartment, but it also requires the infection rate of the susceptible individuals in the past time *t* − *τ* to account for those who receive treatment at the present time *t*. Therefore, we need to keep track of the history of the *S* and *I*_*w*_ compartments, which can be done both analytically and computationally (Smith, 2010). The computational codes for the system dynamics were developed in Matlab and are provided as additional Supplemental Information. For the analysis presented here, since re-infection is precluded, we omit the class (*T*) of individuals who are recovered from infection or effectively treated. This model indicates that with the introduction of a resistant-type infection (either initially or during treatment), a self-sustaining epidemic of resistant infections can occur through direct transmission. A more detailed description of the model is provided in the Supplemental Information.

**Figure 2:**
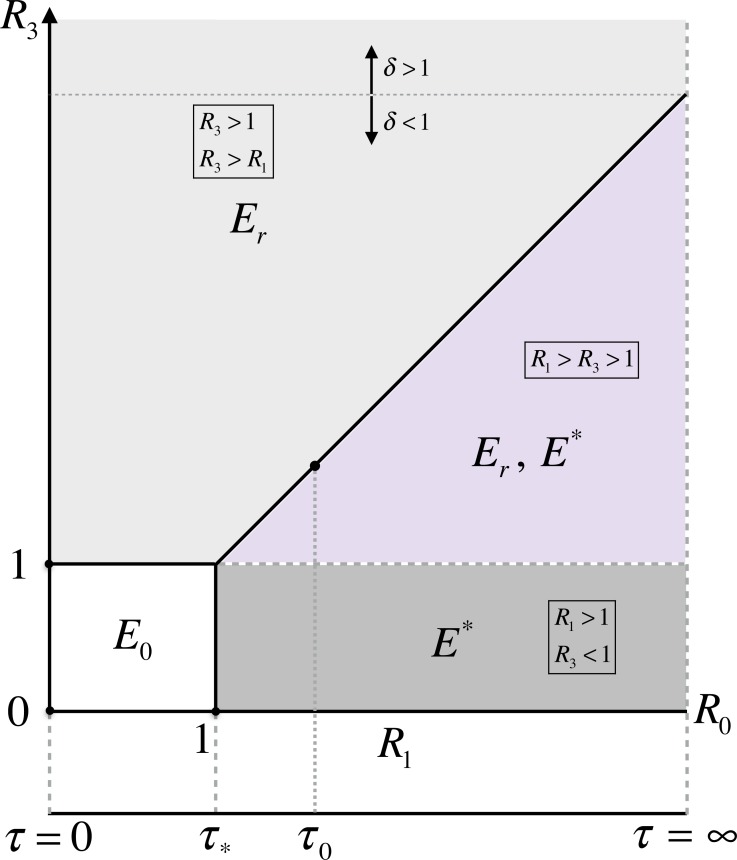
Parameter space for the existence of model equilibria in terms of the reproduction numbers and delay in start of the treatment.

### Equilibria

To characterize the equilibria of the model, we define *R*_0_ = *β*∕(*μ* + *γ*), and apply the next generation method to express the effective reproduction number (*R*_eff_) as the spectral radius of the matrix

\begin{eqnarray*} \left( \begin{array}{@{}ll@{}} \displaystyle {R}_{1}&\displaystyle 0\\ \displaystyle \delta {R}_{2}&\displaystyle {R}_{3} \end{array} \right) , \end{eqnarray*}

where

\begin{eqnarray*}{R}_{1}={R}_{0}\bigl (1-{e}^{-(\mu +\gamma )\tau }\bigr ),\hspace*{20.00003pt}{R}_{2}=q(\tau ){R}_{0}{e}^{-(\mu +\gamma )\tau },\hspace*{20.00003pt}{R}_{3}=\delta {R}_{0}. \end{eqnarray*}

With this notation, *R*_eff_ = max{*R*_1_, *R*_3_}. Each of these quantities provide important information on the spread of disease by each pathogen subtype. Specifically, *R*_1_ represents the number of secondary cases of the wild-type infection generated by an infectious case of the wild-type before developing resistance; *δR*_2_ represents the number of secondary cases of the resistant-type infection generated by an infectious case of the wild-type after developing resistance; and *R*_3_ is the number of secondary cases of the resistant-type infection generated by an infectious case of the resistant-type. In this formulation, *R*_1_ approaches *R*_0_ for large *τ*, and *R*_0_ represents the number of secondary cases generated by a single case of the wild-type infection in the absence of treatment. As illustrated in Fig. 2, we may characterize the existence of the model equilibria using the thresholds of persistence. For simplicity, we describe the equilibria by their infection components of the pathogen subtypes. The infection-free equilibrium *E*_0_ = (0, 0) exists unconditionally. The resistant-type equilibrium ${E}_{r}=(0,\widehat{{I}_{r}})$, at which the wild-type infection is absent, exists only if *R*_3_ > 1. The cotype equilibrium ${E}^{\ast }=({I}_{w}^{\ast },{I}_{r}^{\ast })$ exists whenever both conditions *R*_1_ > 1 and *R*_1_ > *R*_3_ hold.

### Delay-dependent analysis

We focus our analysis on the equilibria when *R*_0_ > 1 to establish a relationship between the delay in start of the treatment and the fraction of population infectious at equilibria. As we will show, this fraction is affected by two key parameters: the probability of developing resistance, and the relative transmission fitness of the resistant-type compared with that of the wild-type.

When the resistant-type emerges with a positive fitness advantage (i.e., *δ* ≥ 1 or equivalently *R*_3_ ≥ *R*_0_), *E*_*r*_ is the only infection equilibrium of the model, at which the fraction of population infectious is independent of the delay in start of the treatment, given by

(2) \begin{eqnarray*}\widehat{{I}_{r}}= \frac{\mu }{\mu +\gamma } \biggl (1- \frac{1}{{R}_{3}} \biggr ).\end{eqnarray*}

If the resistant-type emerges with a negative fitness advantage (i.e., *δ* < 1 or equivalently *R*_3_ < *R*_0_), then there is a critical value *τ*_0_ =  − log(1 − *δ*)∕(*μ* + *γ*) at which *R*_1_(*τ*_0_) = *R*_3_ (Fig. 2). For *τ* < *τ*_0_ (*R*_3_ > *R*_1_ > 1), similar to the case of *δ* ≥ 1, *E*_*r*_ remains the only infection equilibrium of the model.

At *τ* = *τ*_0_, the cotype equilibrium *E*^∗^ emerges and exists along with the resistant-type equilibrium *E*_*r*_ for *τ* ≥ *τ*_0_. We define the fraction of population infectious at the cotype equilibrium as a function of *τ*, and let $G(\tau )={I}_{w}^{\ast }(\tau )+{I}_{r}^{\ast }(\tau )$ represent this function. At *E*^∗^, the value of *G*(*τ*) depends on the probability of developing resistance during treatment in addition to the transmission fitness of the resistance-type. Here we obtain the condition to characterize the behaviour of *G*(*τ*) at *τ*_0_. Taking the implicit derivative of *G*(*τ*) at *τ*_0_ gives

(3) \begin{eqnarray*}{G}^{{^{\prime}}}(\tau ) & = \frac{\mathrm{d}}{\hspace*{2.5pt}\mathrm{d}\tau } \Bigl ({I}_{w}^{\ast }(\tau )+{I}_{r}^{\ast }(\tau )\Bigr )= \frac{\hspace*{2.5pt}\mathrm{d}}{\hspace*{2.5pt}\mathrm{d}\tau } \Biggl ( \frac{{R}_{1}-{R}_{3}}{{R}_{2}} \Biggr ){I}_{r}^{\ast }(\tau )+\Biggl (1+ \frac{{R}_{1}-{R}_{3}}{{R}_{2}} \Biggr ) \frac{\hspace*{2.5pt}\mathrm{d}}{\hspace*{2.5pt}\mathrm{d}\tau } {I}_{r}^{\ast }(\tau )\nonumber\\\displaystyle & =\Biggl ( \frac{{R}_{1}^{{^{\prime}}}{R}_{2}-({R}_{1}-{R}_{3}){R}_{2}^{{^{\prime}}}}{{R}_{2}^{2}} \Biggr ){I}_{r}^{\ast }(\tau )+\Biggl (1+ \frac{{R}_{1}-{R}_{3}}{{R}_{2}} \Biggr ) \frac{\hspace*{2.5pt}\mathrm{d}}{\hspace*{2.5pt}\mathrm{d}\tau } {I}_{r}^{\ast }(\tau ).\end{eqnarray*}

Since ${R}_{1}^{{^{\prime}}}({\tau }_{0})/{R}_{2}({\tau }_{0})=(\mu +\gamma )/q({\tau }_{0})$, we have

\begin{eqnarray*}{G}^{{^{\prime}}}({\tau }_{0})= \frac{(\mu +\gamma )}{q({\tau }_{0})} {I}_{r}^{\ast }({\tau }_{0})+ \frac{\hspace*{2.5pt}\mathrm{d}}{\hspace*{2.5pt}\mathrm{d}\tau } {I}_{r}^{\ast }({\tau }_{0}). \end{eqnarray*}

Taking the derivative of infection component with the resistant-type at *τ*_0_ (see Supplemental Information) gives

\begin{eqnarray*}{G}^{{^{\prime}}}({\tau }_{0})= \frac{\mu }{q({\tau }_{0})} \Biggl ( \frac{1}{\delta } -1\Biggr )\Biggl (q({\tau }_{0})-1+ \frac{1}{{R}_{3}} \Biggr ). \end{eqnarray*}

This implies that the fraction of population infectious at *E*^∗^ decreases if

(4) \begin{eqnarray*}q({\tau }_{0})\lt 1- \frac{1}{{R}_{3}} \end{eqnarray*}

and increases otherwise. As we show in the Supplemental Information, the condition (4) also holds true for the more general case with the inclusion of disease-induced death rate in the *I*_*w*_ and *I*_*r*_ classes. When (4) holds, the start of the treatment with delay *τ* > *τ*_0_ reduces the fraction of population infectious at the cotype equilibrium. However, for sufficiently large *τ*, *G*(*τ*) increases and saturates at

\begin{eqnarray*}{G}_{\infty }=\lim _{\tau \rightarrow \infty }G(\tau )= \frac{\mu }{\mu +\gamma } \Bigl (1- \frac{1}{{R}_{0}} \Bigr ). \end{eqnarray*}

**Figure 3:**
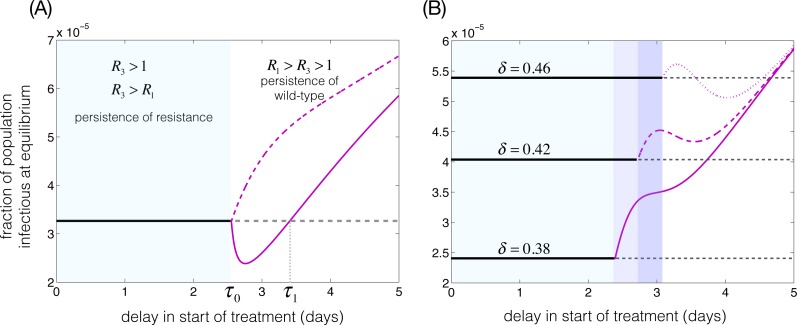
The fraction of population infectious at the equilibria. Parameters are: *R*_0_ = 3, *μ* = 1∕70 year^−1^, and *γ* = 1∕5 day^−1^. (A) *δ* = 0.4, *τ*_0_ = 2.55 days, *τ*_1_ = 3.4 days, *q*(*τ*) = 2*e*^−*aτ*^∕(1 + *e*^−2(*τ*−1)^); with *a* = 1.5 (solid magenta curve) and *a* = 0.5 (dashed magenta curve). (B): *q*(*τ*) = 0.6 for *τ* ≤ *τ*_0_ − 0.06 and *q*(*τ*) = 0.6*e*^−2(*τ*−*τ*_0_+0.06)^ for *τ* > *τ*_0_ − 0.06, with *δ* = 0.38 (solid magenta curve), *δ* = 0.42 (dashed magenta curve), and *δ* = 0.46 (dotted magenta curve). Solid and dashed horizontal lines correspond to the fraction of population infectious at the resistant-type equilibrium.

One can show that *G*(*τ*) < *G*_∞_ for any *τ* (see Supplemental Information), indicating that when *δ* < 1, treatment reduces the population level of infection at the cotype equilibrium, regardless of the probability of developing resistance. We also note that ${G}_{\infty }\gt \widehat{{I}_{r}}$. This suggests that, given (4), there is an intermediate interval for delay in start of the treatment after *τ*_0_, in which the magnitude of infection at the cotype equilibrium reduces below that of the resistant-type equilibrium (Fig. 3A).

When *q*(*τ*_0_) > 1 − 1∕*R*_3_, the magnitude of infection at the cotype equilibrium increases for *τ* > *τ*_0_ and sufficiently close to *τ*_0_. The continual increase in the population level of infection for *τ* > *τ*_0_ depends on the functional form of the probability of resistance development. As shown in our simulations (Fig. 3B), it is possible to reduce the fraction of population infectious at the cotype equilibrium below that of the resistant-type for some intermediate interval of *τ* that remains at positive distance from *τ*_0_. Characterizing *q*(*τ*) and the conditions for the existence of such an intermediate interval is not an easy task, but we show this possibility in our simulations.

When the demographics are omitted (*μ* = 0), the model represents an epidemic scenario without exhibiting any endemic states. In this case, a similar question on the fraction of population infected during the epidemic may be formulated in terms of the epidemic final size (i.e., the total number of infections throughout the epidemic), given by (see Supplemental Information)

\begin{eqnarray*}F= \frac{\gamma \bigl (1-q(\tau ){e}^{-\gamma \tau }\bigr )}{1-{e}^{-\gamma \tau }} \int \nolimits \nolimits _{0}^{\infty }{I}_{w}(t)\hspace*{2.5pt}\mathrm{d}t+\gamma \int \nolimits \nolimits _{0}^{\infty }{I}_{r}(t)\hspace*{2.5pt}\mathrm{d}t. \end{eqnarray*}

**Figure 4:**
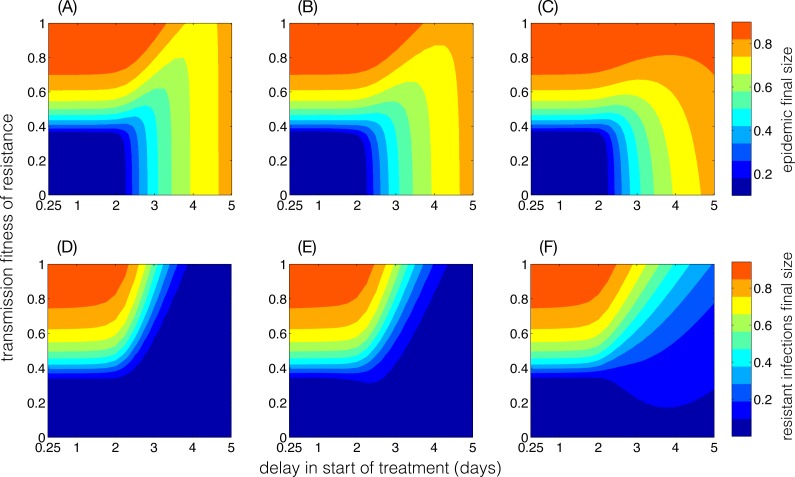
Final size of the epidemic and resistant infections as functions of delay in start of the treatment and the transmission fitness of the resistant-type. Parameters are: *R*_0_ = 3, *γ* = 1∕5 day^−1^, and the functional forms of *q*(*τ*) in (A, D), (B, E) and (C, F) correspond to the red, black, and blue curves illustrated in Fig. S2.

**Figure 5:**
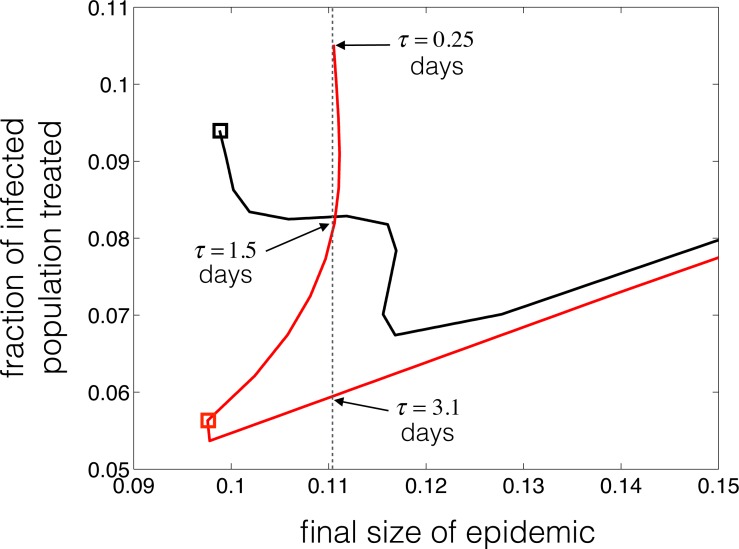
Illustration of the parametric curves (*F*(*τ*), *e*^−*γτ*^*F*(*τ*)) for the epidemic final size and the fraction of infected population treated, with *τ* as an independent variable. Parameters are: *R*_0_ = 2.2, *γ* = 1∕5 day^−1^, *δ* = 0.48, and the functional forms of *q* in black and red curves are given by black and red curves shown in Fig. S2.

Previous work on the population dynamics of drug-resistance emergence without demographics focuses on the fraction of infected population treated to quantify the epidemic size (Lipsitch et al., 2007; Moghadas, 2008; Moghadas et al., 2008; Xiao, Brauer & Moghadas, 2016). Summarizing the outcomes, these models demonstrate that there is an optimal treatment coverage at which the epidemic final size is minimized. The uniqueness of such optimal coverage has not been proven (Xiao, Brauer & Moghadas, 2016). Our simulations for the model presented here show that, for a given transmission fitness of the resistant-type, different delays in start of the treatment may lead to the same epidemic final size depending on the probability of resistance development at the time of treatment initiation (Figs. 4 and 5). This effectively means that the minimum epidemic final size may be achieved with different population levels of treatment.

## Simulation Results

To illustrate the theoretical results, based on the competitive dynamics between the wild-type and resistant-type, we simulated the cotype equilibrium by varying the delay in start of the treatment within the average infectious period of 1∕*γ* = 5 days (Table 1). We assumed a reproduction number *R*_0_ = 3 in the absence of treatment, and fixed the transmission fitness of resistant-type at *δ* = 0.4, giving 1 − 1∕*R*_3_ = 0.1667. Figure 3A shows the curves of equilibria for two different functional forms of *q*(*τ*) = 2*e*^−*aτ*^∕(1 + *e*^−2(*τ*−1)^), as illustrated in Fig. S1. The solid curve represents the emergence of the cotype equilibrium at *τ*_0_ = 2.55, where *a* = 1.5 and *q*(*τ*_0_) = 0.0418 satisfies the condition (4), and therefore the fraction of population infectious at the cotype equilibrium reduces below that of the resistant-type equilibrium in the interval (*τ*_0_, *τ*_1_) (Fig. 3A). The dashed curve corresponds to the case where *a* = 0.5 and *q*(*τ*_0_) = 0.5348 disobeys the condition (4).

**Table 1:** Parameter values used for simulations extracted from the published literature (Alexander et al., 2007; Moghadas, 2008; Moghadas et al., 2008; Xiao, Brauer & Moghadas, 2016).

Parameter	Baseline values (range)	Comments
*μ*	1/70 per year	assumed
*R*_0_	2.2, 3 (>1)	the value of *R*_0_ used for simulations are within the estimated ranges for influenza epidemics and pandemics
*δ*	variable [0–1]	varied in simulations
*γ*	0.2 per day	the value of *γ* corresponds to an average infectious period of five days within the estimated range for influenza infection
*τ*	variable [0–1∕*γ*]	varied in simulations for the length of infectious period
*q*(*τ*)	variable	the probability of developing resistance during treatment was determined from the functional form of *q*(*τ*)

We further simulated the cotype equilibrium with the probability of developing resistance that has the functional form

\begin{eqnarray*}q(\tau )= \left\{ \begin{array}{@{}ll@{}} \displaystyle 0.6\hspace*{10.00002pt}&\displaystyle \text{if}\tau \lt {\tau }_{0}-0.06\\ \displaystyle 0.6{e}^{-2(\tau -{\tau }_{0}+0.06)}\hspace*{10.00002pt}&\displaystyle \text{if}\tau \geq {\tau }_{0}-0.06 \end{array} \right. \end{eqnarray*}

with *τ*_0_ = 2.55. Figure 3B shows the behaviour of the cotype equilibrium for different values of *δ*, and represents the possibility of an intermediate interval of *τ* in which the magnitude of infection at the cotype equilibrium is reduced below that of the resistant-type equilibrium (i.e., dotted curve for the case of *δ* = 0.46).

In the absence of demographics (*μ* = 0), we simulated the model with parameter values described in Table 1 to determine the final size of the epidemic with different functional forms of *q*(*τ*). Figure 4 shows heatmaps for the epidemic final size and the final size of the resistant infections as functions of delay in start of the treatment and transmission fitness of resistance. Clearly, the patterns of epidemic final size are affected by *q*(*τ*), which also determines how early the competitive balance is shifted in favour of the resistant-type. The functional forms of *q*(*τ*) are shown in Fig. S2. We observed that for a given transmission fitness *δ*, it is possible to obtain the same epidemic final size with several delays in start of the treatment. For *R*_0_ = 2.2 and *δ* = 0.48, Fig. 5 illustrates the change in the final size of epidemic with the fraction of infected population that is treated. The particular forms of *q*(*τ*) (Fig. S2) indicate that if delay in start of treatment exceeds a certain amount of time, the same final size may be achieved with different treatment levels (Fig. 5, black and red curves). For the black curve, the lowest final size is achieved when the treatment starts at the onset of infectious period, which corresponds to the highest fraction of infected individuals being treated. However, using a different functional form of *q*(*τ*) (Fig. S2, red curve), we observed that the lowest final size occurs with *τ* = 2.75 days delay in start of the treatment (within the infectious period), which corresponds to a lower treatment level of the infected population. Importantly, the lowest epidemic final sizes in black and red curves are approximately the same (represented by black and red boxed), but occurs at different delays in start of the treatment and at different fractions of infected population treated.

## Discussion

The emergence and spread of drug-resistance have been studied in a number of epidemic and endemic models of infectious diseases (Lipsitch et al., 2007; Moghadas, 2008; Handel, Longini Jr & Antia, 2007; Legros & Bonhoeffer, 2016; Mills, Cohen & Colijn, 2013). A key parameter in these models is the probability of resistance development at the host level, which has been considered to be independent of the time for start of the treatment. In this study, we developed a simple two-subtype disease transmission model to study the population dynamics of drug-resistance spread when the probability of developing resistance is a function of delay in the treatment initiation. We discussed the transient and equilibrium states of the resistant and wild pathogen subtypes in short-term (epidemic) and long-term (endemic) scenarios. In contrast to previous work (D’Agata et al., 2008), our results show that an early initiation of treatment may not necessarily be the optimal strategy for preventing the emergence and spread of drug-resistance (Fig. 5). Our results also show that, depending on the probability of resistance development at the host level and the relative transmission fitness of the resistant-type at the population level, the minimum infection state of the system at equilibrium for both pathogen subtypes may occur with considerable delay in start of the treatment during the infectious period (Fig. 3). This is not necessarily consistent with current treatment practices, in which the management of infection and severe outcomes in patients takes precedence over the possible evolution and spread of drug-resistance under the selection pressure of drugs.

The probability of developing resistance at the host level is affected by several factors, including the frequency with which the resistant mutants arise, compensatory mutations that restore impaired fitness of resistance, the efficacy of drugs in preventing pathogen growth, the concentration of drugs, and the level and strength of the host immune responses (White, 2004). The time for start of the treatment can have a substantial influence on the underlying processes by which these factors are characterized. At the population level, the interplay between these factors manifests itself in the rise and fall of resistance (Lipsitch, 2001), making the identification of optimal treatment regimens extremely difficult to achieve in order to simultaneously minimize the incidence of disease and limit resistance emergence and spread (Colijn & Cohen, 2015; Day & Read, 2016). As has been demonstrated in recent studies, it is therefore important to develop multi-scale models that integrate both within-host infection dynamics and between-host disease transmission (Legros & Bonhoeffer, 2016). Our theoretical framework and analyses here are not meant to address the question of optimal treatment strategies in either the host or population level, but rather to underscore the complexity of drug-resistance dynamics in both the epidemic and endemic disease states.

In the model presented here, we have made several simplifying assumptions. We assumed that those who are effectively treated (without developing resistance) do not contribute to disease transmission following the initiation of treatment. This effect can be adjusted in the model by tuning other parameters such as the infectious period. We assumed the same infectious period for both the resistance-type and wild-type infections. Our distinct theoretical analyses show that similar analytical results can still be achieved, and we therefore chose this simplification. We also assumed that all infectious individuals will receive treatment with delay *τ* after the onset of infectious period, if they have not recovered until time *τ*. Realistically, in several diseases (e.g., influenza), a sizeable fraction of infectious individuals may not receive treatment for a number of reasons such as mild illness or asymptomatic infection. While the coverage of treatment can be included in the model as an independent parameter, we note that the effect of reduced treatment coverage can also be adjusted through a longer infectious period. Our model is based on a single treatment regimen which is assumed to be ineffective against resistant-type infection. In some infectious diseases (e.g., influenza, HIV/AIDS, tuberculosis) there are a number of drugs that may be used sequentially or in combination to prevent or manage resistance in treated patients. Although we have not considered multi-drug treatment, the effect of drugs on resistant-type infection may be included in the probability of developing resistance in our model. While these assumption can be relaxed and the model be extended to consider the aforementioned factors, we believe this simple structure underscores the importance of the probability of resistance development with delay in start of the treatment, and clearly demonstrates the complex dynamics of drug-resistance spread in the population. Finally, our results echo the take-home message drawn from some recent studies that the management of drug-resistance requires theoretical frameworks that combine the two scales of micro and macro dynamics to not only minimize the short-term impact of disease on the population, but also to address the long-term epidemiological consequences of the pathogen evolutionary responses under the pressure of drug treatment.

##  Supplemental Information

10.7717/peerj.2817/supp-1 Supplemental Information 1 Revised Supplementary Material

10.7717/peerj.2817/supp-2 Figure S1 Probability of developing resistance

10.7717/peerj.2817/supp-3 Figure S2 Probability of developing resistance with delay

10.7717/peerj.2817/supp-4 Figure S3 Illustration of parametric curves

10.7717/peerj.2817/supp-5 Supplemental Information 2 Matlab code The Matlab code to find the numerical solutions of the full model for a given set of parameters

10.7717/peerj.2817/supp-6 Supplemental Information 3 Matlab Code for Fig. 3A of the main text

10.7717/peerj.2817/supp-7 Supplemental Information 4 Matlab Code for Fig. 3B of the main text

10.7717/peerj.2817/supp-8 Supplemental Information 5 Matlab Code for Fig. 4 of the main text

10.7717/peerj.2817/supp-9 Supplemental Information 6 Matlab Code for Fig. 5 of the main text

10.7717/peerj.2817/supp-10 Supplemental Information 7 Matlab Code for Fig. S2 of the Supplemental Information

## Additional Information and Declarations

### Data Availability

The following information was supplied regarding data availability: The research in this article did not collect or analyse any raw data from the biological field. All sources of parameters from other studies have been cited in the manuscript.
