# Supplementary material for: Population dynamics of epidemic and endemic states of drug-resistance emergence in infectious diseases
Source: PeerJ. 2017 Jan 10;5:e2817. doi: 10.7717/peerj.2817 (PMC5228518; doi:10.7717/peerj.2817)
Supplement: Supplemental Information 1 [file peerj-05-2817-s001.pdf]

Supplemental Information

Population Dynamics of Epidemic and Endemic States of Drug-Resistance Emergence in Infectious Diseases

Diána Knipl,[1] Gergely Röst,[2] Seyed M. Moghadas[3,*]

[1]*Department of Mathematics, University College London, London WC1E 6BT, United Kingdom*

[2]*Bolyai Institute, University of Szeged, 6720 Szeged, Hungary*

[3]*Agent-Based Modelling Laboratory, York University, Toronto, ON M3J 1P3, Canada*

∗ corresponding author (moghadas@yorku.ca)

This supplemental information provides details of a more general model with additional theoretical arguments and simulations supporting the results presented in the main text.

Details of the full model

Recruitment of individuals into the population is included through the compartment S at a constant rate μ, and therefore the model does not include any additional sources of infection from outside the population. We include treatment into our model with the delay τ for all infectious individuals who have not been recovered. Drug-resistance may develop as a result of treatment failure, which occurs with the probability $q(\tau)$ as a function of delay in start of treatment. New infections are modelled by mass-action

incidence with the baseline transmission rate β for the wild-type infection. Direct transmission of the resistant-type infection occurs at the rate $\delta\beta$, where δ denotes the relative transmission fitness of the resistant-type compared with the wild-type. We assume the same recovery rate γ for both the wild-type and resistant-type infections.

Since treatment is ineffective against resistant infection, individuals infected with the resistant-type in the I_r class remain infectious until recovery or death. However, individuals infected with the wild-type in the I_w class will be treated at time τ (before recovery or death), and are successfully treated with the probability of $1 - q(\tau)$. We assume that these individuals are non-infectious, and therefore move to a non-infectious (recovered) class T. A fraction $q(\tau)$ of treated individuals will develop resistance and move to the I_r class. The class T accumulates recovered individuals as well as those who are successfully treated from the wild-type infection. Here, we consider a more general case of the model presented in the main text where the average lifetime may differ between infectious and healthy individuals as a result of disease-induced death. We represent these two rates with μ and μ_d for infectious and healthy individuals, respectively. Thus the infection dynamics in time t is governed by the following system of differential equations :

$$\begin{aligned}
S'(t) &= \mu - \beta S(t)[I_w(t) + \delta I_r(t)] - \mu S(t),\\
I_w'(t) &= \beta S(t) I_w(t) - \beta S(t-\tau) I_w(t-\tau) e^{-(\mu_d+\gamma)\tau} - \gamma I_w(t) - \mu_d I_w(t),\\
I_r'(t) &= \delta\beta S(t) I_r(t) + q(\tau)\beta S(t-\tau) I_w(t-\tau) e^{-(\mu_d+\gamma)\tau} - \gamma I_r(t) - \mu_d I_r(t),\\
T'(t) &= (1-q(\tau))\beta S(t-\tau) I_w(t-\tau) e^{-(\mu_d+\gamma)\tau} + \gamma[I_w(t) + I_r(t)] - \mu T(t).
\end{aligned} \tag{1}$$

Note that the term $-\beta S(t-\tau) I_w(t-\tau) e^{-(\mu_d+\gamma)\tau}$ corresponds to the treatment of individuals who have become infectious at time $t - \tau$ and receive treatment at time t, assuming that they have not been removed from the I_w class upon recovery or death. Since treatment is ineffective against the resistant-type infection, we have omitted the treatment term in the I_r class, while they are treated without moving out of this class due to treatment. Without loss of generality, we assume that the initial size of the total population is $S(0) + I_w(0) + I_r(0) + T(0) = 1$. Since the dynamics of infection is independent of variable T, we have dropped the equation for T in the model analysis discussed in the main text and detailed here.

Equilibria analysis

In model (1), the reproduction numbers take the form:

$$R_1 = R_0\big(1 - e^{-(\mu_d+\gamma)\tau}\big), \qquad \delta R_2 = \delta q(\tau) R_0 e^{-(\mu_d+\gamma)\tau}, \qquad R_3 = \delta R_0.$$

When $R_3 > 1$, there is a resistant-type equilibrium $E_r = (0, \widehat{I}_r)$, where

$$\widehat{I}_r = \frac{\mu}{\mu_d+\gamma}\Big(1 - \frac{1}{R_3}\Big).$$

If $\delta \geq 1$ ($R_3 \geq R_0$), E_r is the only equilibrium at which the disease is present. For $\delta < 1$, E_r is the only infection equilibrium if $\tau < \tau_0$, where

$$\tau_0 = -\frac{\log(1-\delta)}{\mu_d+\gamma}$$

and $R_1(\tau_0) = R_3$. At τ_0, the cotype equilibrium $E^* = (I_w^*, I_r^*)$ emerges whose infection components are

$$I_r^* = \frac{\mu}{\mu_d+\gamma}\left(\frac{R_2(R_1-1)}{R_0(R_1-R_3)+R_2R_3}\right), \quad I_w^* = \frac{\mu}{\mu_d+\gamma}\left(\frac{(R_1-R_3)(R_1-1)}{R_0(R_1-R_3)+R_2R_3}\right).$$

In this case, for $\tau > \tau_0$, the model has both resistant-type and cotype equilibria. Let $G(\tau) = I_w^*(\tau) + I_r^*(\tau)$ denote the fraction of population infectious at the cotype equilibrium (as a function of τ) for $R_0 > R_1 > R_3 > 1$. It is easy to see that

$$G_\infty = \lim_{\tau\to\infty} G(\tau) = \frac{\mu}{\mu_d+\gamma}\Big(1 - \frac{1}{R_0}\Big).$$

We claim that $G(\tau) < G_\infty$ for any $\tau > \tau_0$. To prove our claim, we show

$$\frac{(R_1-1)(R_1+R_2-R_3)}{R_0(R_1-R_3)+R_2R_3} < 1 - \frac{1}{R_0}$$

which is equivalent to

$$R_0(R_1-1)(R_1+R_2-R_3) < (R_0-1)[R_0(R_1-R_3)+R_2R_3]. \tag{2}$$

Expanding both sides and using $R_2R_3 < R_0R_2$, it is sufficient to show

$$R_0R_1R_2 + R_0R_1^2 + R_0^2R_3 < R_0^2R_1 + R_0R_2R_3 + R_0R_1R_3.$$

Substituting from the expression for R_1, R_2, and R_3 in terms of R_0, we get

$$\begin{aligned}(1-e^{-(\mu_d+\gamma)\tau})q(\tau)e^{-(\mu_d+\gamma)\tau} + (1-e^{-(\mu_d+\gamma)\tau})^2 + \delta <& \delta q(\tau)e^{-(\mu_d+\gamma)\tau} \\ &+ (1+\delta)(1-e^{-(\mu_d+\gamma)\tau}).\end{aligned}$$

Rearranging this inequality gives $(1-e^{-(\mu_d+\gamma)\tau}-\delta)(1-q(\tau))e^{-(\mu_d+\gamma)\tau} > 0$, which holds if and only if $\delta < 1-e^{-(\mu_d+\gamma)\tau}$. Since $R_3 < R_1$, the inequality (2) holds and our claim is proven.

We now provide the details of calculation to obtain the inequality (4) in the main text when $\delta < 1$. The derivative of the infection component of the resistant-type is

$$\frac{\mathrm{d}}{\mathrm{d}\tau}I_r^*(\tau_0) = \frac{\mu}{q(\tau_0)R_3}\Big[R_2 - R_0\Big(1-\frac{1}{R_3}\Big)\Big]. \tag{3}$$

Using the equation (3) in the main text, we have

$$\begin{aligned}G'(\tau_0) &= \frac{(\mu_d+\gamma)}{q(\tau_0)}\Big[\frac{\mu}{\mu_d+\gamma}\Big(1-\frac{1}{R_3}\Big)\Big] + \frac{\mu}{q(\tau_0)R_3}\Big[R_2 - R_0\Big(1-\frac{1}{R_3}\Big)\Big] \\ &= \frac{\mu}{q(\tau_0)R_3}\Big[R_2 - (R_3-1)\Big(\frac{1}{\delta}-1\Big)\Big].\end{aligned}$$

Since $R_1(\tau_0) = R_3$, it follows that $e^{-(\mu_d+\gamma)\tau_0} = 1-\delta$, and therefore $R_2 = q(\tau_0)R_0(1-\delta)$. Thus,

$$\begin{aligned}G'(\tau_0) &= \frac{\mu}{q(\tau_0)}\Big[\frac{R_2}{R_3} - \Big(1-\frac{1}{R_3}\Big)\Big(\frac{1}{\delta}-1\Big)\Big] \\ &= \frac{\mu}{q(\tau_0)}\Big[q(\tau_0)\Big(\frac{1}{\delta}-1\Big) - \Big(1-\frac{1}{R_3}\Big)\Big(\frac{1}{\delta}-1\Big)\Big] \\ &= \frac{\mu}{q(\tau_0)}\Big(\frac{1}{\delta}-1\Big)\Big(q(\tau_0)-1+\frac{1}{R_3}\Big).\end{aligned}$$

The above theoretical arguments also apply to the model presented in the main text in which $\mu = \mu_d$, from which the condition (4) in the main text is derived.

Final size for the epidemic case

For the epidemic scenario with $\mu = \mu_d = 0$, we let $\mathscr{I}_w$ represent the total number of individuals infected with the wild-type throughout the epidemic. The probability of receiving treatment is $e^{-\gamma\tau}$, and therefore the total number of the wild-type infections recovered before receiving treatment is given by

$$(1 - e^{-\gamma\tau})\mathscr{I}_w = \gamma \int_0^\infty I_w(t)\,\mathrm{d}t \tag{4}$$

Given the probability $q(\tau)$ for developing resistance, the total number of the wild-type infections who are effectively treated is $(1 - q(\tau))e^{-\gamma\tau}\mathscr{I}_w$. Thus, the total number of the wild-type infections who recover without developing resistance is

$$\begin{aligned}&\gamma(1 - q(\tau))\frac{e^{-\gamma\tau}}{1 - e^{-\gamma\tau}} \int_0^\infty I_w(t)\,\mathrm{d}t + \gamma \int_0^\infty I_w(t)\,\mathrm{d}t \\ &= \frac{\gamma(1 - q(\tau)e^{-\gamma\tau})}{1 - e^{-\gamma\tau}} \int_0^\infty I_w(t)\,\mathrm{d}t.\end{aligned} \tag{5}$$

Adding this to the total number of individuals recovered from the resistant-type infection gives the total number of infection presented by F in the main text.

Probability of developing resistance

In this section, we provide the functional forms of the probability of developing resistance. Figure S1 represents $q(\tau)$ for the results of cotype equilibrium presented in Figure 3(A) of the main text. The probability of developing resistance is given by

$$q(\tau) = \frac{2e^{-a\tau}}{1 + e^{-2(\tau-1)}}, \quad \text{for } a > 0.$$

We used $a = 1.5$ (Figure S1, solid curve) and $a = 0.5$ (Figure S1, dashed curve) for simulations presented in Figure 3(A) of the main text.

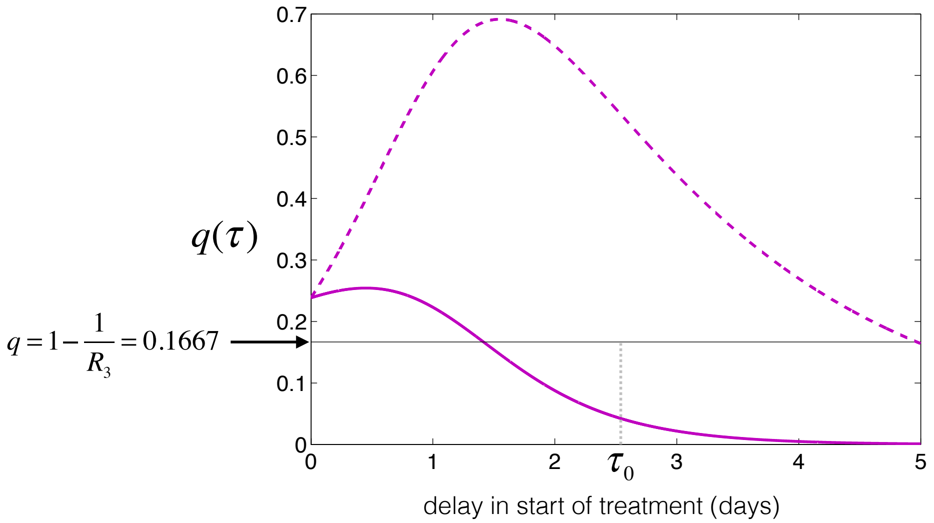

Figure S1: Probability of developing resistance as a function of delay in start of treatment. Parameters are: $R_0 = 3$, $\mu = \mu_d = 1/70$ year^{-1}, $\gamma = 1/5$ day^{-1}, $\delta = 0.4$, $\tau_0 = 2.55$ days, and $\tau_1 = 3.4$ days. The probability of developing resistance is given by $q(\tau) = 2e^{-a\tau}/(1 + e^{-2(\tau-1)})$ with $a = 1.5$ (Solid magenta curve) and $a = 0.5$ (dashed magenta curve).

To illustrate the possible behaviour of the cotype equilibrium presented in Figure 3(B) of the main text, we used the following functional form of $q(\tau)$:

$$q(\tau) = \begin{cases} 0.6 & \text{if } \tau \leq \tau_0 - 0.06 \\ 0.6e^{-2(\tau-\tau_0+0.06)} & \text{if } \tau > \tau_0 - 0.06 \end{cases}$$

To simulate the model for epidemic final size without demographics, we used the following functional forms of the probability of developing resistance:

1. Figure 4(A) of the main text

$$q(\tau) = \frac{15e^{-2\tau}}{1 + e^{-2(\tau-2)}}, \quad \text{corresponding to the red curve in Figure S2;}$$

2. Figure 4(B) of the main text

$$q(\tau) = \frac{2e^{-\tau}}{1 + e^{-5(\tau-1.5)}}, \quad \text{corresponding to the black curve in Figure S2;}$$

3. Figure 4(C) of the main text

$$q(\tau) = \frac{0.25}{1+e^{-2(\tau-2)}}, \quad \text{corresponding to the blue curve in Figure S2.}$$

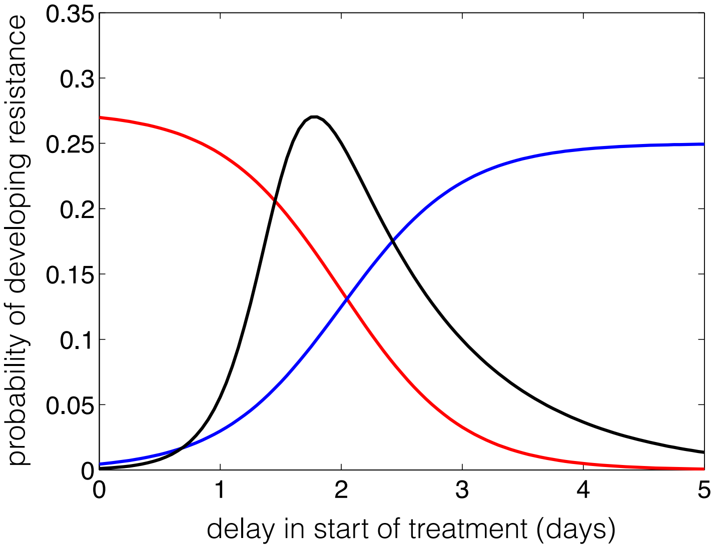

Figure S2: Probability of developing resistance with delay in start of treatment, corresponding to the epidemic final sizes presented in Figure 4 of the main text. Colour curves correspond to $q(\tau) = 15e^{-2\tau}/(1+e^{-2(\tau-2)})$ (red; Figure 4A of the main text); $q(\tau) = 2e^{-\tau}/(1+e^{-5(\tau-1.5)})$ (black; Figure 4B of the main text); and $q(\tau) = 0.25/(1+e^{-2(\tau-2)})$ (blue; Figure 4C of the main text).

For each curve presented in Figure S2, we plotted the final size of epidemic and the fraction of infected population treated in a pairwise coordinate for different transmission fitness of the resistant-type (Figure S3). The corresponding curves show that for some functional forms of $q(\tau)$, it is possible to achieve the same final size with different fractions of infected population treated, which is achieved with different delays in start of the treatment during the infectious period. Two specific cases for different functional forms of $q(\tau)$ and $\delta = 0.48$ are illustrated in Figure 5 of the main text.

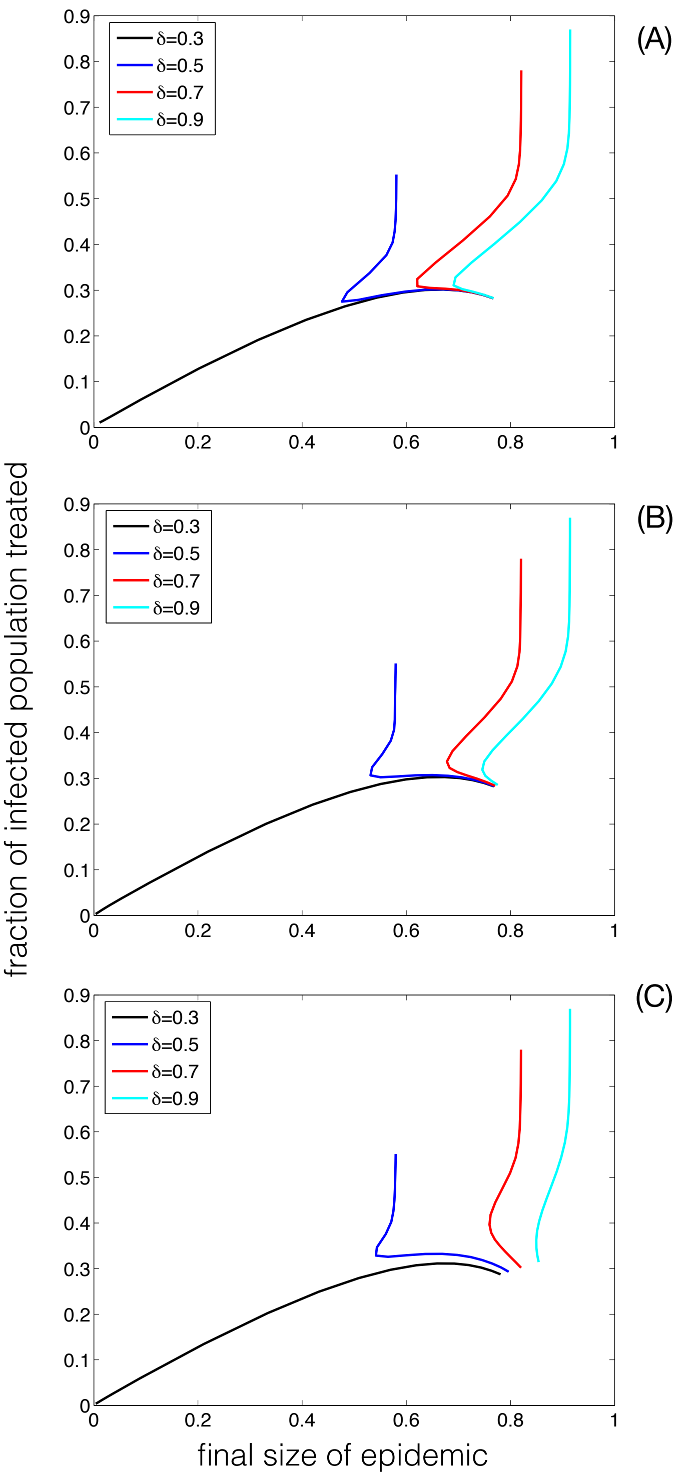

Figure S3: Illustration of parametric curves $(F(\tau), e^{-\gamma\tau}F(\tau))$ for the epidemic final size and the fraction of infected population treated, with τ as an independent variable. Parameters are: $R_0 = 3$, $\gamma = 1/5$ day^{-1} when τ varies between 0 and $1/\gamma = 5$. The probability of developing resistance has the functional forms (A): $q(\tau) = 15e^{-2\tau}/(1 + e^{-2(\tau-2)})$; (B): $q(\tau) = 2e^{-\tau}/(1 + e^{-5(\tau-1.5)})$; and (C) $q(\tau) = 0.25/(1 + e^{-2(\tau-2)})$.
