# Supplementary figures and images for: Population dynamics of epidemic and endemic states of drug-resistance emergence in infectious diseases
Source: PeerJ. 2017 Jan 10;5:e2817. doi: 10.7717/peerj.2817 (PMC5228518; doi:10.7717/peerj.2817)

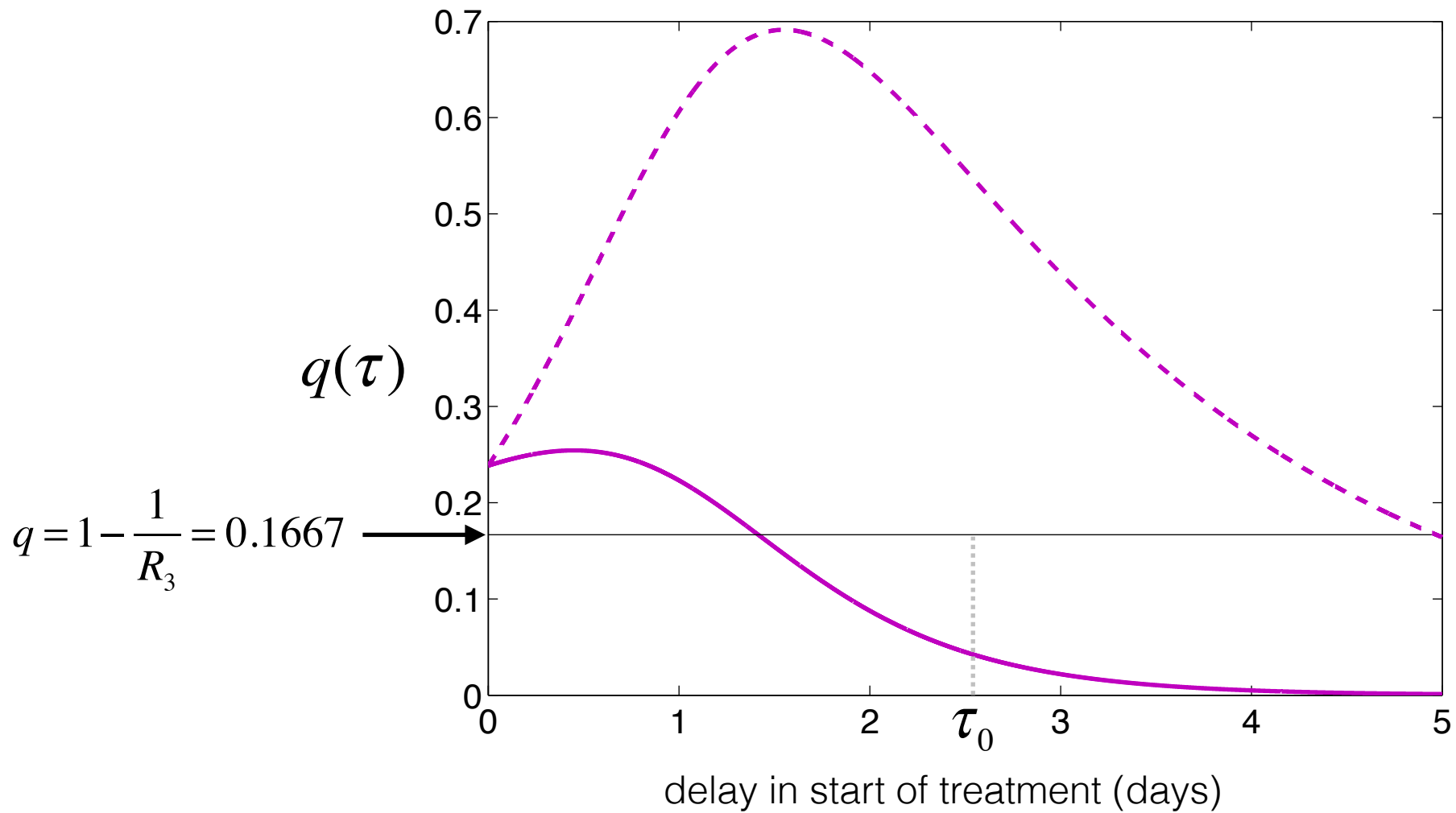

Supplement: Figure S1 [file peerj-05-2817-s002.pdf]

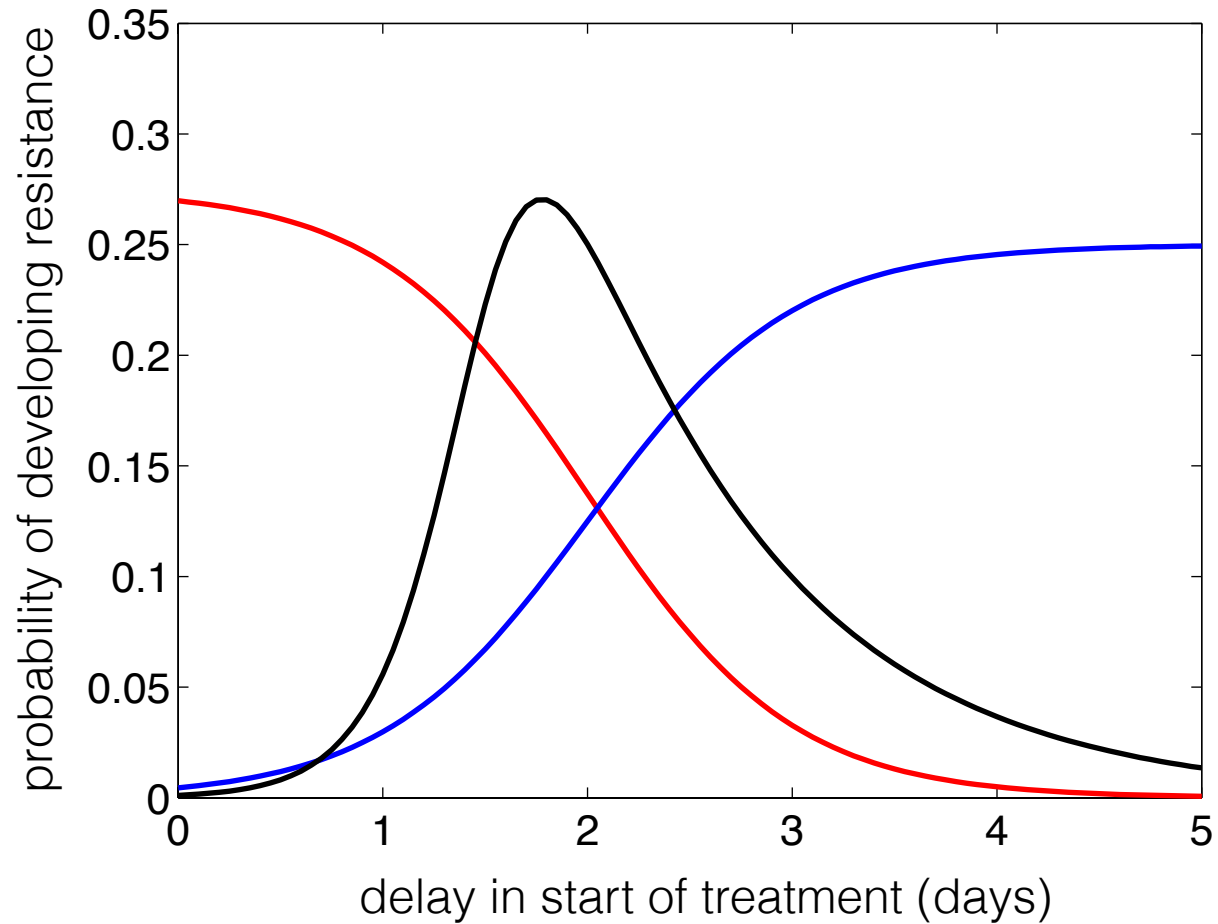

Supplement: Figure S2 [file peerj-05-2817-s003.pdf]

fraction of infected population treated

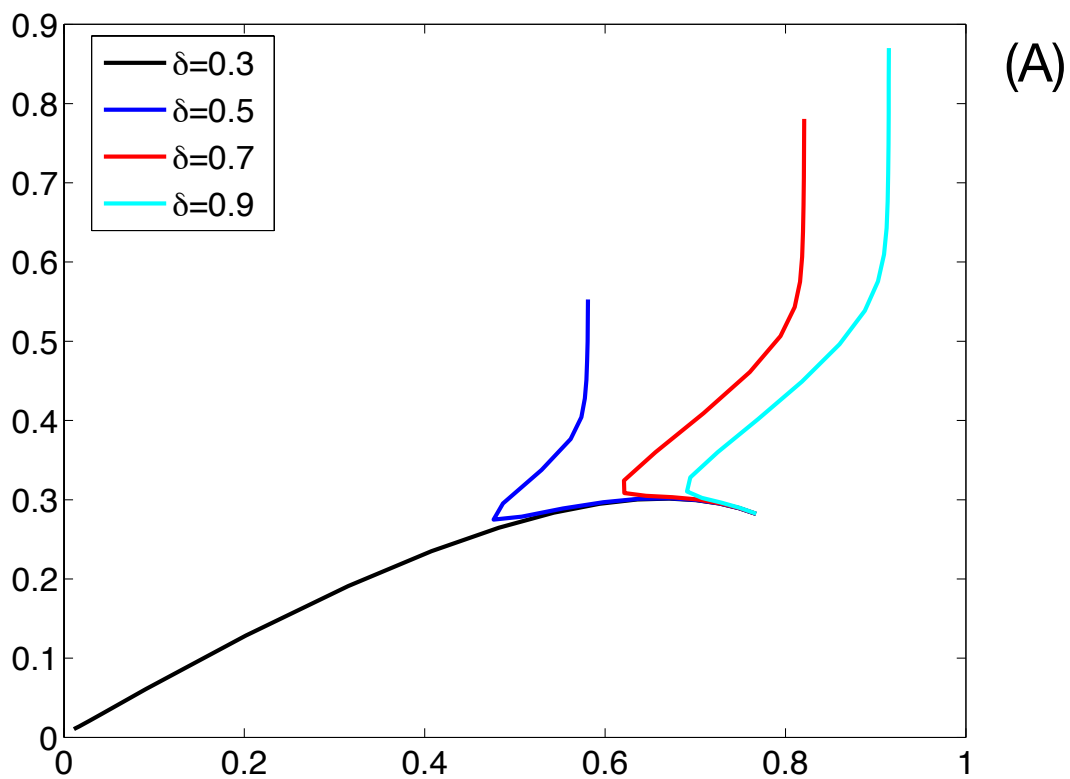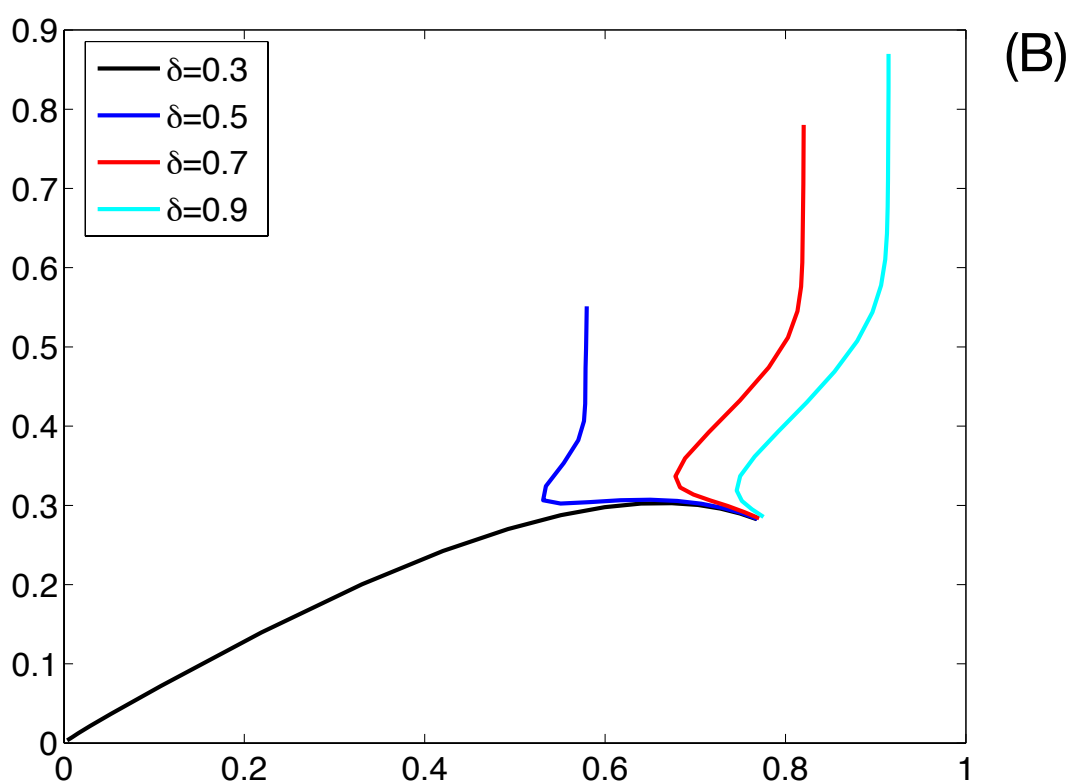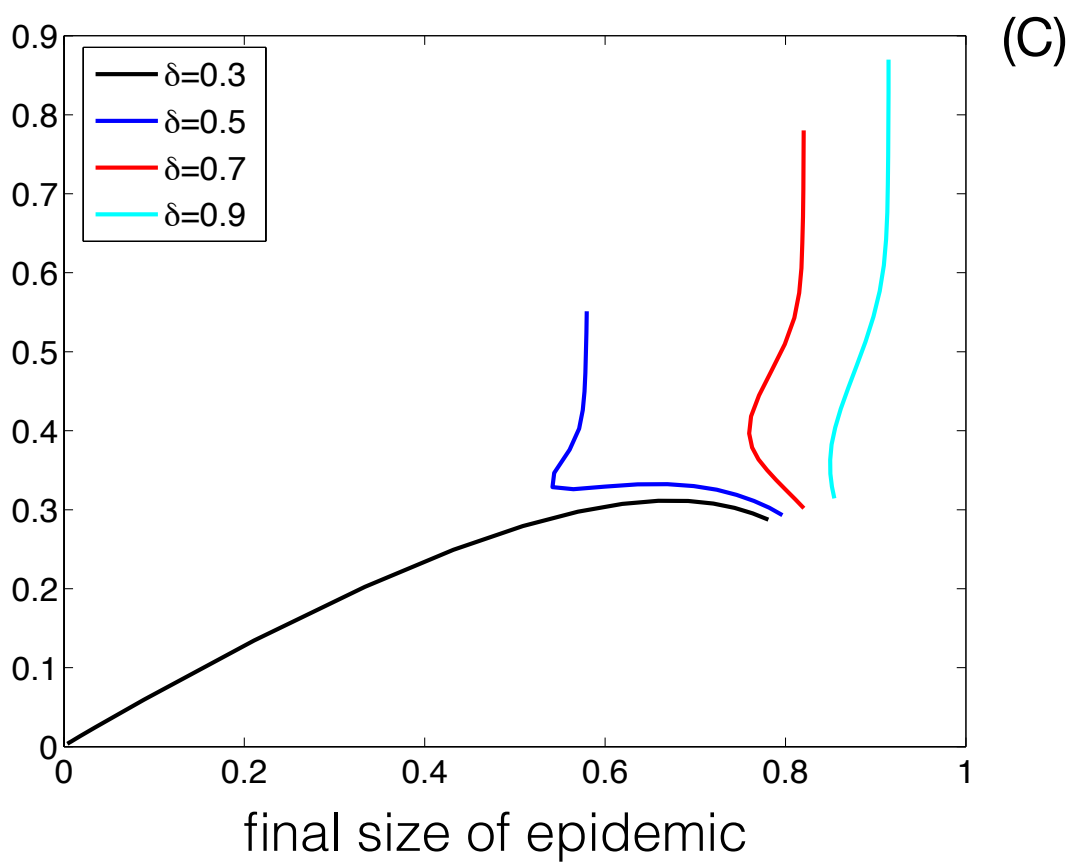

Supplement: Figure S3 [file peerj-05-2817-s004.pdf]
